# Regional left atrial wall fibrosis and recurrence after atrial fibrillation ablation

**DOI:** 10.1186/1532-429X-18-S1-P200

**Published:** 2016-01-27

**Authors:** Erik T Bieging, Alan Morris, Joshua Cates, Nassir F Marrouche

**Affiliations:** 1 Cardiology, University of Utah, Salt Lake City, UT USA; 2 CARMA Center, University of Utah, Salt Lake City, UT USA; 3 SCI Institute, University of Utah, Salt Lake City, UT USA

## Background

Left atrial (LA) wall fibrosis has been demonstrated to play a major role in the pathogenesis and treatment response of atrial fibrillation (AF). The DECAAF study demonstrated that quantification of LA fibrosis by delayed-enhancement MRI (DE-MRI) predicts outcomes after AF catheter ablation. Currently, a number of catheter ablation methods targeting specific regions of the LA are used, including pulmonary vein isolation and posterior wall debulking. We investigate the role of spatial distribution of fibrosis in the LA on outcomes after catheter ablation.

## Methods

The DECAAF study was a prospective, multicenter, observational cohort study in which patients underwent DE-MRI prior to AF catheter ablation, and were followed for AF recurrence for up to 475 days after a 90-day blanking period, with methods previously published. In this study, 254 patients enrolled in the DECAAF study had regions of the LA wall manually segmented into 4 walls: anterior, posterior, lateral, and septal. The ostia of the pulmonary veins and the left atrial appendage were excluded. The posterior wall was defined as the region between the pulmonary vein ostia, extending to the posterior mitral annulus and LA floor. The anterior wall was defined as the region bound by the anterior mitral annulus, the LA floor, the interatrial septum, the superior pulmonary vein ostia, and the LA appendage. The lateral wall was defined by the left pulmonary vein ostia, the LA appendage, and the lateral mitral annulus. The septal wall was defined by the interatrial septum. LA fibrosis score was calculated from the DE-MRI images for each wall using a previously described thresholding method. Repeated-measures one-way ANOVA was used to compare fibrosis score between walls. Univariate Cox regression was performed using the fibrosis score for each wall, with AF recurrence as the outcome. Harrell's C-statistic was used to compare the predictive power of each score.

## Results

Fibrosis score was highest on the lateral wall (37.0%), followed by the posterior wall (29.6%), anterior wall (27.8%), and septal wall (20.6%). All pairwise comparisons were statistically significant (*p* < 0.001) except for anterior vs. posterior wall (*p* = 0.65). Univariate Cox regression showed that the fibrosis score of each individual wall was predictive of AF recurrence after catheter ablation (all *p* < 0.001). The predictive power was similar for each of the four walls: the posterior wall C-statistic was 0.63, anterior wall 0.63, lateral wall 0.62, and septal wall 0.60. None of the individual wall fibrosis scores predicted recurrence better than total fibrosis score, which had a C-statistic of 0.66.

## Conclusions

Fibrosis quantified by DE-MRI differentially affects the walls of the LA, affecting the lateral wall most, followed by the posterior and anterior walls. However, fibrosis score in any wall predicts recurrence after AF catheter ablation, suggesting that LA wall fibrosis may be important regardless of its location.

**Figure 1:**
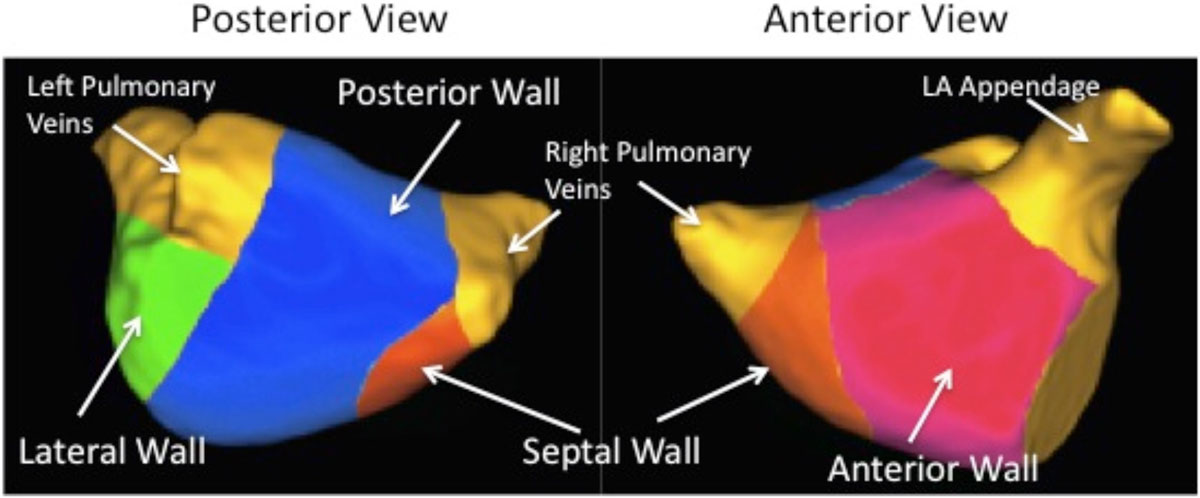
**The LA was manually segmented into four walls: posterior, anterior, lateral, and septal, as shown**.

**Figure 2:**
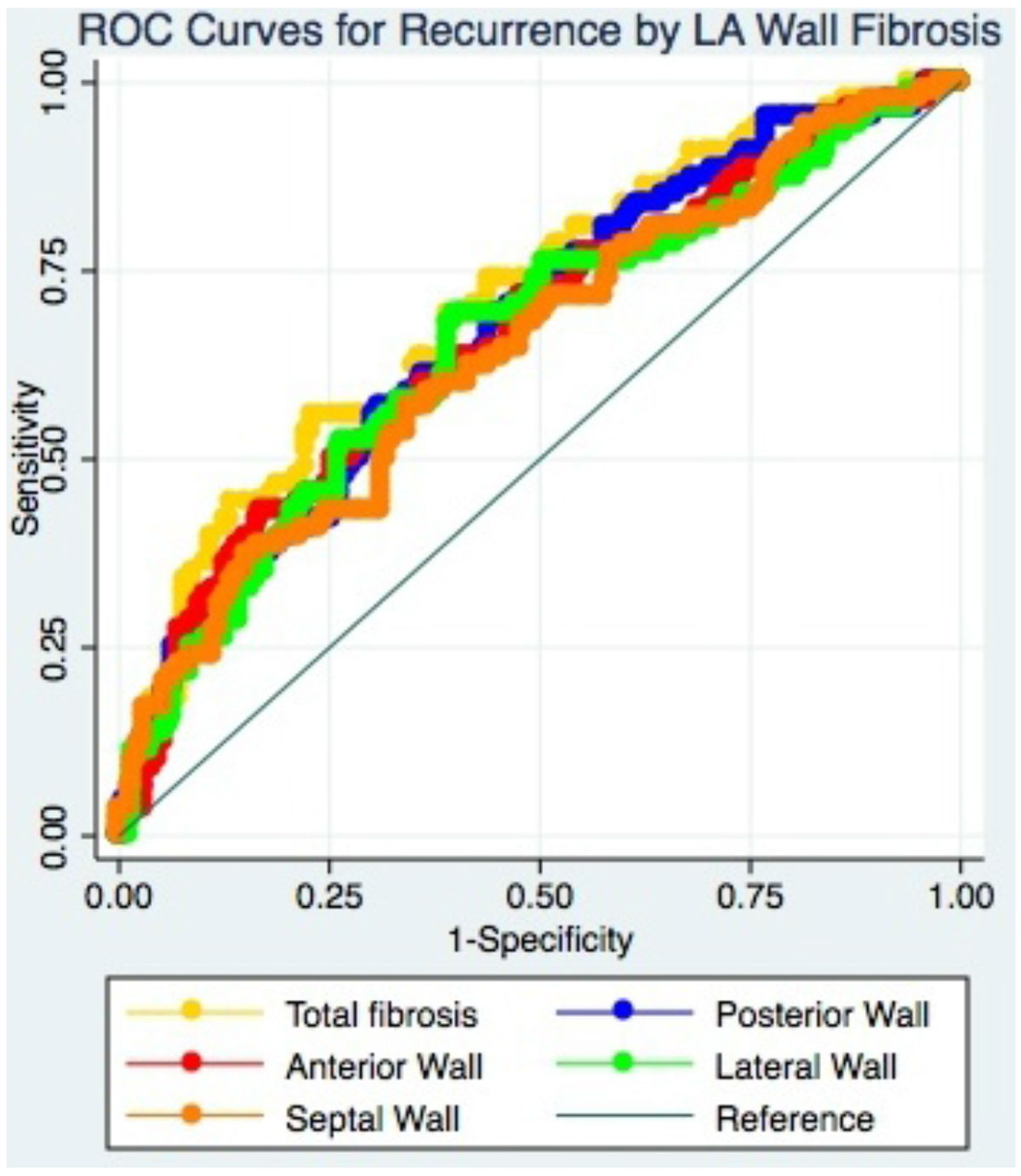
**ROC curves for prediction of AF recurrence based on fibrosis score for each wall, as well as the total fibrosis score, are shown**. All walls showed similar predictive power as demonstrated by overlapping ROC curves.

